# Molecular and Neurodevelopmental Benefits to Children of Closure of a Coal Burning Power Plant in China

**DOI:** 10.1371/journal.pone.0091966

**Published:** 2014-03-19

**Authors:** Deliang Tang, Joan Lee, Loren Muirhead, Ting Yu Li, Lirong Qu, Jie Yu, Frederica Perera

**Affiliations:** 1 Department of Environmental Health Sciences, Columbia Center for Children's Environmental Health, Mailman School of Public Health, Columbia University, New York, New York, United States of America; 2 Department of Pediatrics, Chongqing Medical University, Chongqing, China; Federal University of Rio de Janeiro, Brazil

## Abstract

Polycyclic aromatic hydrocarbons (PAH) are major toxic air pollutants released during incomplete combustion of coal. PAH emissions are especially problematic in China because of their reliance on coal-powered energy. The prenatal period is a window of susceptibility to neurotoxicants. To determine the health benefits of reducing air pollution related to coal-burning, we compared molecular biomarkers of exposure and preclinical effects in umbilical cord blood to neurodevelopmental outcomes from two successive birth cohorts enrolled before and after a highly polluting, coal-fired power plant in Tongliang County, China had ceased operation. Women and their newborns in the two successive cohorts were enrolled at the time of delivery. We measured PAH-DNA adducts, a biomarker of PAH-exposure and DNA damage, and brain-derived neurotrophic factor (BDNF), a protein involved in neuronal growth, in umbilical cord blood. At age two, children were tested using the Gesell Developmental Schedules (GDS). The two cohorts were compared with respect to levels of both biomarkers in cord blood as well as developmental quotient (DQ) scores across 5 domains. Lower levels of PAH-DNA adducts, higher concentrations of the mature BDNF protein (mBDNF) and higher DQ scores were seen in the 2005 cohort enrolled after closure of the power plant. In the two cohorts combined, PAH-DNA adducts were inversely associated with mBDNF as well as scores for motor (p = 0.05), adaptive (p = 0.022), and average (p = 0.014) DQ. BDNF levels were positively associated with motor (p = 0.018), social (p = 0.001), and average (p = 0.017) DQ scores. The findings indicate that the closure of a coal-burning plant resulted in the reduction of PAH-DNA adducts in newborns and increased mBDNF levels that in turn, were positively associated with neurocognitive development. They provide further evidence of the direct benefits to children's health as a result of the coal plant shut down, supporting clean energy and environmental policies in China and elsewhere.

## Introduction

Polycyclic aromatic hydrocarbons (PAH) are a group of compounds formed from the incomplete combustion of coal and other organic material. PAH are ubiquitous in the environment, present in outdoor and indoor air from coal combustion, diesel and other motor vehicle emissions, tobacco smoking and cooking of food [1], [2]. In China, rapid economic expansion coupled with escalating fossil fuel-based energy production has resulted in massive amounts of air pollution. Specifically, coal-fired power plants produce over 70 percent of China's electricity, and new power plants being designed are also run on coal, which contributes to unsafe levels of toxic contaminants, including PAH [3], [4], [5].

Inhalation of airborne PAH leads to formation of DNA adducts, which are considered a valid marker of the biologically effective dose of PAH, reflecting individual variation in metabolism of PAH and DNA repair [6]. PAH are carcinogenic and neurotoxic. Previous studies by the Columbia Center for Children's Environmental Health (CCCEH) in New York City, Krakow and the present Tongliang cohort have shown that the developing fetus is more susceptible than the adult to PAH-DNA adduct formation. They have also shown that prenatal PAH exposure is associated with adverse impacts on child neurodevelopment, including developmental delay, reduced IQ, behavioral problems in childhood [7], [8], [9], [10], [11], [12] and reduced intelligence scores later in childhood [10], [12].

In Tongliang County, the high pollutant concentrations in ambient air prompted the government to shut down the local power plant in May of 2004 to significantly improve community health [4], [13]. This action, announced in advance, provided a unique opportunity to compare air monitoring, biomarker and health outcome data in two successive cohorts of children with and without prenatal exposure to emissions from the coal-fired power plant. In partnership with Chongqing Children's Hospital, the CCCEH carried out two prospective cohort studies between 2002 and 2005, pre-plant shutdown and post-plant shutdown, respectively. We previously reported evidence from the 2002 cohort that prenatal exposure to PAH adversely affected the neurodevelopment of children on the GDS at age 2 [11] and that the later cohort had significantly lower levels of PAH-DNA adducts in cord blood [9]. Additionally, the significant associations observed in the 2002 cohort between PAH-DNA adducts and adverse outcomes on the GDS at age 2 were no longer seen in the 2005 cohort [9].

Here we have analyzed levels of BDNF and their relationship to both adducts and developmental outcomes in the two cohorts. BDNF is critical for neurological survival and cognitive development of the central nervous system (CNS) [14], [15] and is the most widely distributed neurotrophin [16]. BDNF is first released as the proBDNF precursor before being cleaved into mBDNF [14]. Regulation of BDNF depends on its site of release: postsynaptic release is regulated by a Ca^2+^ influx through ionotropic glutamate receptors and voltage gated Ca^2+^ channels [17]; release from presynaptic sites is also dependent on mobilizing Ca^2+^ influx from intracellular stores [17], [18]. Following release, BDNF binds to two different transmembrane proteins: tropomyosin- related TrkB receptor and neurotrophin receptor p75, with higher affinity for the TrkB receptor [17]. Specifically, the binding of TrkB receptor triggers three signaling cascades that ultimately phosphorylate and activate the cAMP responsive element binding protein (CREB) transcription factor to encourage genes transcription essential for neuronal development and synaptic plasticity [17], [19].

Because BDNF and TrkB are expressed in the hippocampus to reinforce and stabilize synaptic connections, BDNF has been widely recognized as a key regulator in long-term potentiation (LTP), one of several phenomena underlying synaptic plasticity, learning and memory [16], [20]. LTP has been divided into early LTP and late LTP, where early LTP (characterized as short term potentiation) is shorter lasting and depends on the modification and translocation of proteins [21], and late LTP lasts several days and depends on de novo protein synthesis [20]. BDNF is an active mediator of neuronal processes in both the developing and mature brain, promoting differentiation, growth, and survival of neurons during development [22]. For late LTP, studies have shown that higher endogenous levels of BDNF are required to activate multiple signaling cascades that may act concertedly to regulate downstream cellular effects for memory formation and maintenance [23]. Therefore, in the mature brain, memory formation and development are associated with increased levels of BDNF and TrkB activation [24].

The goal of this paper is to determine whether mBDNF can provide a potential risk marker in assessing the neurodevelopment effects of exposure to PAH during fetal development, and in documenting the benefits of a regulatory intervention. Because previous studies have shown adverse effects of lower BDNF levels on learning and memory processes [24], we hypothesized that increased levels of PAH-DNA adducts would be associated with decreased levels of mBDNF that, in turn, would mediate the effects on neurodevelopmental outcomes determined by the DQ.

## Methods

The overall approach used in this investigation and the comparison of the two cohorts with respect to adducts and DQ scores have been presented elsewhere [9].

### Ethics Statement

This study was approved by the Institutional Review Board of Columbia University. All subjects gave informed written consent by completing a form approved by both the Columbia University IRB and by Chongqing Medical University.

### Population

The city of Tongliang has a population of approximately 810,000 and is situated in a small basin approximately 3 km in diameter [3]. A coal-fired power plant located south of the town center operated during the dry season from 1 December to 31 May each year prior to 2004 in order to compensate for insufficient hydroelectric power during that time period. This plant was the principal source of local air pollution because in 1995 nearly all domestic heating and cooking units were converted to natural gas, motor vehicles were not a major pollution source, and there were no major coal-burning sources within 20 km of the city [4], [13]. In May 2004, the power plant was closed and replaced by the national grid system of electrical energy.

### Study subjects

In the 2002 cohort, as previously reported [9], the subjects were 150 nonsmoking mothers and their newborns enrolled between 4 March 2002 and 19 June 2002 at four hospitals in Tongliang: the Tongliang County Hospital, the Traditional Chinese Medicine Hospital, the Tongliang Maternal Children's Health Hospital, and the Bachuan Hospital. In the 2005 cohort, the subjects were 158 children born at the same hospitals from 2 March 2005 to 23 May 2005 and recruited and studied using the same methods. The women were selected using a screening questionnaire when they checked in for delivery. Eligibility criteria included current nonsmoking status, ≥20 years of age, and residence within 2.5 km of the Tongliang power plant. All but one eligible woman agreed to enter the study. The demographic characteristics of the two cohorts are presented in Table 1.

**Table 1:** Demographic and Exposure Characteristics of the Cohorts.

		2002 Cohort	2005 Cohort
Mother's Age, years*		25.18±3.152, N = 110	27.909±4.585, N = 107
Mother's Education (%)	<High School	48 (43.64%)	59(55.14%)
	≥High School	62 (56.36%)	48(44.86%)
Gestational Age, days		277.35±11.27, N = 110	276.692±9.188, N = 107
Sex of Newborn, % female		56 (50.91%), N = 110	48(44.86%), N = 107
Birth Head Circumference, cm*		33.77±1.151, N = 110	34.130±1.283, N = 106
Infant's Birth Weight, g		3346.64±398.03, N = 110	3378.505±399.653, N = 107
Infant's Birth Length, cm		50.38±1.690, N = 110	50.212±1.635, N = 104
Heavy Metals	Cord Lead (mg/dL)	3.60±1.59	3.74±1.50
	Cord Mercury (ppb)	6.67±4.43	6.61±2.77
PAH-DNA Adducts, adducts/10^8^ nucleotides*		0.324±0.139, N = 110	.204±.081, N = 107
ETS(hours/day)		0.293±0.586, N = 110	0.297±0.539, N = 107
Income**	Total N	77	107
	<10000	2	22
	10001 to 20000	13	52
	20001 to 30000	30	17
	30001 to 40000	22	6
	40001 to 50000	7	5
	50001 to 60000	3	3
	60001 to 70000	0	0
	>70000	0	2
BDNF, μg/dL*		752.87±463.71, N = 108	1266.57±619.77, N = 107
Gesell Scores	Average	99.42±10.74, N = 110	100.3±7.157, N = 107
	Motor	97.53±11.47, N = 110	97.83±7.821, N = 107
	Adaptive	98.71±14.90, N = 110	101.18±10.96, N = 107
	Language*	102.1±12.83, N = 110	100.47±9.777, N = 107
	Social	99.40±11.79, N = 110	101.83±6.808, N = 107

*significant difference between the two cohorts at alpha  = 0.05 level, using t-test.

** significant difference between the two cohorts at alpha  = 0.05 level, using chi-square test.

### Personal interview

A 45-min questionnaire was administered by a trained interviewer after delivery. The questionnaire elicited demographic information, lifetime residential history (location of birth and duration of residence), history of active and passive smoking (including number of household members who smoke), occupational exposure, medication use, alcohol consumption during each trimester of pregnancy, and consumption of PAH-containing meat (frequency of eating fried, broiled, or barbecued meat during the last 2 weeks). Socioeconomic information related to income and education was also collected.

### Biological sample collection and biomarker analysis

40–60 mL of umbilical cord blood was collected at delivery and 10 mL of maternal blood (10 mL) was collected within 1 day postpartum. Samples were transported to the field laboratory at the Tongliang County Hospital immediately after collection for processing. Blood samples, the buffy coat, packed red blood cells, and plasma were separated and stored at −70°C. B[a]P is widely used as a representative of PAH because concentrations of individual PAH in the urban setting are highly intercorrelated. Therefore B[a]P –DNA adducts were analyzed as a proxy for PAH-DNA adducts [25]. Details of laboratory methods for analyzing B[a]P-DNA adducts have been described[11]. Immunoassays for plasma levels of BDNF were performed using the BDNF E_max_ ImmunoAssay System (Promega) according to the manufacturer's instruction.

### Measurement of child neurodevelopment using GDS

Two-year-old children in the cohort were administered the Chinese version of the standardized GDS for 0- to 3-year-old children adapted to the Chinese population by the Chinese Pediatric Society from the department of Pediatric Psychiatry in Xinghua Hospital in Shanghai, China. Each child is assigned a DQ in each of four areas: motor, adaptive, language, and social. The standardized mean (±SD) of the DQ is 100±15; a score <85 indicates developmental delay [26]. Testing was conducted by physicians in the same group who were certified in the GDS to maximize reliable assessment and valid interpretation, minimizing both inter-examiner and intra-examiner variability.

### Statistical Analysis

We compared characteristics of the two cohorts using the t-test or chi-square test. Multiple linear regression and logistic regression were used to test the relationships among cord adducts, BDNF, and DQ. Covariates in the regression models were selected based on previous findings and included cord blood lead, cord mercury, environmental tobacco smoke (ETS), mother's education, mother's age, gestational age, and gender. Cord adducts were calculated on the original scale, while cord lead was dichotomized at the median of detectable lead in the multiple linear regression models. BDNF values were log transformed to normalize data and remove outliers. DQ scores were handled on the original scale. Cord blood BDNF levels of newborns in the two cohorts were compared using the Mann-Whitney test. We explored whether BDNF was a mediator of the relationship between PAH-DNA adducts and DQ scores by comparing the change in the beta coefficient of cord adducts in two regression models, with and without BDNF as a variable.

## Results

The demographics, exposure, and developmental characteristics of each cohort are provided and compared in Table 1. There were no significant differences between the cohorts with respect to these characteristics except in mother's age and income level. The mean level of mBDNF was significantly higher in the 2005 cohort as compared to the 2002 cohort (1266.56 pg/ml vs. 752.87 pg/ml, P<0.05). The mean birth head circumference of the 2005 infants was significantly greater than that of the 2002 cohort (33.766 cm vs. 34.130 cm, P<0.05). The mean PAH-DNA cord adduct level was 0.324 adducts/10^8^ nucleotides in the 2002 cohort and 0.204 adducts/10^8^ nucleotides in the 2005 cohort, also a significant difference (P<0.05). BDNF levels were also significantly higher in the 2005 cohort, with a mean of 752.871 μg/dL in the 2002 cohort compared to 1266.568 μg/dL in the 2005 cohort (P<0.05). Although the differences were not statistically significant in all areas, DQ scores were consistently higher in the 2005 cohort than the 2002 cohort for motor, adaptive, social, and average DQ (Table 1).

In cord blood, PAH-DNA adducts were modestly but significantly inversely correlated with BDNF (r = −0.233, p<0.01, N = 269). The results of multiple regression analysis of the association between biomarkers and DQ scores are shown in Table 2 for levels of PAH-DNA adducts and BDNF protein separately. The regression coefficients (betas) represent the change in DQ score per unit increase in cord adducts or per log unit BDNF. Combining the two cohorts, a unit increase in cord adducts was negatively associated with average (β = −12.113, p = 0.014), motor (β = −10.699, p = 0.050, and adaptive (β = −16.472, p = 0.022) DQ scores after adjusting for cord blood lead, cord mercury, ETS, mother's education, mother's age, gestational age, and gender. A log unit increase in log transformed BDNF level was positively associated with average (β = 2.496, p = 0.017), motor area (β = 2.117, p = 0.018), and social area DQ (β = 3.222, p = 0.001) scores, after adjusting for covariates including cord PAH-DNA adducts, prenatal ETS, cord lead, cord mercury, gender, gestational age, mother's education, mother's age and income. BDNF was deemed to be a potential mediator variable as the betas displayed a change of over 10% after including BDNF as a covariate in the model with adducts as the independent variable and DQs as the dependent variable: the change in average DQ was 21.5%; in motor DQ 33.6%; in adaptive DQ 18.2%; and in social DQ, 37.3%.

**Table 2:** Regression Analysis for DQ and DNA-Adducts and BDNF.

	Average	Motor	Adaptive	Language	Social
**Adducts** 1	−12.113, p = .014	−10.699, p = .050	−16.472, p = .022	−11.679, p = .057	−9.544, p = .078
**(N = 215)**	(−21.786, −2.440)	(−21.409, .010)	(−30.545, −2.398)	(−23.720, .363)	(−20.180, 1.093)
**BDNF** 2	2.496, p = .017	2.117, p = .018	1.844, p = .086	.368, p = .518	3.222, p = .001
**(N = 207)**	(.454, 4.539)	(.467, 4.965)	(−.377, 5.604)	(−1.73, 3.416)	(1.694, 6.068)

1 Adjusting for cord lead (Ln), cord mercury (Ln), ETS (hours/day), mother's education, mother's age, gestational age and gender.

2 Adjusting for income, cord lead (Ln), cord mercury (Ln), ETS (hours/day), mother's education, mother's age, and gestational age.

## Discussion

As hypothesized, comparison of the two cohorts in Tongliang, China, before and after closure of the local power plant, has provided additional evidence of significant measurable benefits on children's development. As previously reported, we found a significant reduction in mean PAH-DNA adduct levels (0.204 adducts/10^8^ nucleotides in the 2005 cohort compared to 0.324 adducts/10^8^ nucleotides in the 2002 cohort). Also, as reported previously, PAH-DNA adducts in cord blood were significantly associated with decrements in motor and language areas, along with overall DQ score in the 2002 cohort [11], consistent with previous evidence that prenatal exposure to B[a]P produces neurodevelopmental effects in the offspring of study animals [27] as well as in children at age 3 years in a New York City cohort [28].

Further, the 2005 cohort had a significantly higher average mBDNF level compared to the 2002 cohort (1266.56 pg/ml vs. 752.87 pg/ml, P<0.05). As PAH-DNA cord adducts were inversely correlated with BDNF levels (r = −0.233, p<0.01), we hypothesized that reduction in BDNF levels as a result of prenatal PAH exposure may have contributed to the adverse neurocognitive effects in the 2002 cohort. Overall, DQ scores were positively associated with BDNF levels. Although the associations were not statistically significant, motor, adaptive, social area, and the average scores were consistently higher in the 2005 cohort compared to the 2002 cohort. Language DQ scores were significantly higher in the 2002 cohort; however language is the least reliable measure for cognitive development because environmental enrichment, such as more parental interaction time, has been shown to reverse potential language deficits [29]. Moreover, our exploratory analyses suggest that BDNF may mediate in part the association between cord adducts and neurocognitive outcomes (average, motor, adaptive and social DQ scores).

The adverse developmental effects observed in the 2002 cohort are consistent with PAH being a maternal contaminant that crosses the placenta to affect neurodevelopment and cognitive function [30]. Furthermore, we have reported that prenatal PAH exposure adversely affects cognitive development [10], [12], [28]. This paradigm of prenatal PAH-induced neurotoxic effects is consistent with results from animal studies, which explained the relationship between downregulation of the gene encoding the receptor tyrosine kinase, MET, a target of B[a]P, which mediates neuronal process growth and synapse formation [31]. In another study, in utero exposure to aerosolized B[a]P altered the expression profile of genes involved in synaptogenesis such as Sp4 transcription factor, as well as glutamate homeostasis and metabolism of reactive oxygen intermediates [32].

These results are consistent with findings of Toledo-Rodriguez et al. that prenatal exposure to ETS (which contains PAH) was associated with increased methylation of the BDNF exon and down-regulation of BDNF[33], and reports of dysfunction in the BDNF system and compromised cognitive function in adult life from fetal exposure to unfavorable intrauterine conditions [34]. This paper is the first to assess BDNF and cognitive development with respect to prenatal exposure to PAH. The mechanisms by which PAH-related reduction of BDNF may cause child developmental problems have not been determined but recent studies have pointed to several potential mechanistic pathways: alteration in the conversion of proBDNF to mBDNF which influences levels of signaling proteins involved in LTP; disruption of activation of TrkB; and induction of methylation changes in BDNF.

First, urban air pollution has been shown to increase levels of plasminogen activator (PA) inhibitor [35], a serine protease necessary to cleave plasminogen into its active form of plasmin [36], [37]. Tissue PA (tPA) is involved in cleaving proBDNF to mBDNF. Defects in tPA-dependent cleavage of proBDNF to mBDNF may downregulate mBDNF, resulting in impaired LTP [36], [37]. Second, the effects of BDNF are believed to be mediated by TrkB, which follows receptor tyrosine kinase activation to initiate three major signaling pathways: phospholipase Cγ (PLCγ), phosphatidylinositol 3-kinase (PI3K), and cascade governed by extracellular signal-regulated kinases (ERK) [38]. Experimentally, prenatal stress decreases hippocampal mBDNF expression and subsequent TrkB- regulated signaling cascades for LTP [39]. The absence of TrkB forces BDNF to selectively bind to p75, resulting in death of the hippocampal neurons [40]. Similarly, higher levels of endogenous proBDNF and low levels of mBDNF were observed in prenatally stressed rats, which influenced synaptic plasticity later in life [37]. Finally, BDNF expression is also regulated by the dynamic methyl CpG binding protein 2 (MeCP2), which may function as a molecular linker between DNA methylation, chromatin remodeling, and ultimately gene repression by recruiting and interacting with histone deacetylases (HDACs) and transcription repressor mSin3A [41], [42]. Experimentally MeCP2 has been shown to bind to methylated CpG sequences in the BDNF promoter III [43] and in BDNF exon IV promoter to induce chromatin compaction and repress gene expression [41]. Prenatal exposure to maternal smoking was associated with enhanced DNA methylation in BDNF exon IV that may lead to long-term deregulation of BDNF [33]. Thus, mBDNF expression is regulated by changes in DNA methylation patterns that have previously been associated with levels of ambient air pollution and prenatal tobacco smoke exposure.

Strengths of the present study are the prospective cohort design, the ability to control for confounders, and the measurement of PAH-DNA adducts as a biological dosimeter for environmental exposure. Additionally, mBDNF is a mechanistically relevant measure of preclinical response/potential risk. A limitation is that we were not able to adjust for postnatal exposure to PAH. However, in this population the major source of PAH was the coal-burning plant emissions prior to its closure.

This study provides insight into the relationship between PAH, PAH-DNA adducts, BDNF, and developmental outcomes. Further studies are needed to determine whether specific enrichment environments can mitigate the damage caused by prenatal environmental exposure to PAH. In conclusion, this provides further evidence that the power plant shutdown in Tongliang, China in May of 2004 directly benefited the health and development of children.

## References

[1] Lijinsky W (1991) The formation and occurrence of polynuclear aromatic hydrocarbons associated with food. Mutat Res 259: 251–261. 2017211 10.1016/0165-1218(91)90121-2

[2] Bostrom CE, Gerde P, Hanberg A, Jernstrom B, Johansson C, et al (2002) Cancer risk assessment, indicators, and guidelines for polycyclic aromatic hydrocarbons in the ambient air. Environ Health Perspect 110 Suppl 3451–488. 12060843 10.1289/ehp.110-1241197 PMC1241197

[3] Millman A, Tang D, Perera FP (2008) Air pollution threatens the health of children in China. Pediatrics 122: 620–628. 18762533 10.1542/peds.2007-3143

[4] (2004) The WADE economic model: China, in World Survey of Decentralized Energy. (WADE) World Alliance for Decentralized Energy.

[5] Zhang J, Mauzerall DL, Zhu T, Liang S, Ezzati M, et al (2010) Environmental health in China: progress towards clean air and safe water. Lancet 375: 1110–1119. 20346817 10.1016/S0140-6736(10)60062-1 PMC4210128

[6] Godschalk RW, Van Schooten FJ, Bartsch H (2003) A critical evaluation of DNA adducts as biological markers for human exposure to polycyclic aromatic compounds. J Biochem Mol Biol 36: 1–11. 12542969 10.5483/bmbrep.2003.36.1.001

[7] Jedrychowski W, Whyatt RM, Camann DE, Bawle UV, Peki K, et al (2003) Effect of prenatal PAH exposure on birth outcomes and neurocognitive development in a cohort of newborns in Poland. Study design and preliminary ambient data. Int J Occup Med Environ Health 16: 21–29. 12705714

[8] Perera F, Li TY, Lin C, Tang D (2012) Effects of prenatal polycyclic aromatic hydrocarbon exposure and environmental tobacco smoke on child IQ in a Chinese cohort. Environ Res 114: 40–46. 22386727 10.1016/j.envres.2011.12.011

[9] Perera F, Li TY, Zhou ZJ, Yuan T, Chen YH, et al (2008) Benefits of reducing prenatal exposure to coal-burning pollutants to children's neurodevelopment in China. Environ Health Perspect 116: 1396–1400. 18941584 10.1289/ehp.11480 PMC2569101

[10] Edwards SC, Jedrychowski W, Butscher M, Camann D, Kieltyka A, et al (2010) Prenatal exposure to airborne polycyclic aromatic hydrocarbons and children's intelligence at 5 years of age in a prospective cohort study in Poland. Environ Health Perspect 118: 1326–1331. 20406721 10.1289/ehp.0901070 PMC2944097

[11] Tang D, Li TY, Liu JJ, Zhou ZJ, Yuan T, et al (2008) Effects of prenatal exposure to coal-burning pollutants on children's development in China. Environ Health Perspect 116: 674–679. 18470301 10.1289/ehp.10471 PMC2367664

[12] Perera FP, Li Z, Whyatt R, Hoepner L, Wang S, et al (2009) Prenatal airborne polycyclic aromatic hydrocarbon exposure and child IQ at age 5 years. Pediatrics 124: e195–202. 19620194 10.1542/peds.2008-3506 PMC2864932

[13] Chow JC, Watson JG, Chen LW, Ho SS, Koracin D, et al (2006) Exposure to PM2.5 and PAHs from the Tong Liang, China epidemiological study. J Environ Sci Health A Tox Hazard Subst Environ Eng 41: 517–542. 16779929 10.1080/10934520600564253

[14] Numakawa T, Suzuki S, Kumamaru E, Adachi N, Richards M, et al (2010) BDNF function and intracellular signaling in neurons. Histol Histopathol 25: 237–258. 20017110 10.14670/HH-25.237

[15] Cohen-Cory S, Kidane AH, Shirkey NJ, Marshak S (2010) Brain-derived neurotrophic factor and the development of structural neuronal connectivity. Dev Neurobiol 70: 271–288. 20186709 10.1002/dneu.20774 PMC2893579

[16] Lu Y, Christian K, Lu B (2008) BDNF: a key regulator for protein synthesis-dependent LTP and long-term memory? Neurobiol Learn Mem 89: 312–323. 17942328 10.1016/j.nlm.2007.08.018 PMC2387254

[17] Cunha C, Brambilla R, Thomas KL (2010) A simple role for BDNF in learning and memory? Front Mol Neurosci 3: 1. 20162032 10.3389/neuro.02.001.2010 PMC2821174

[18] Balkowiec A, Katz DM (2002) Cellular mechanisms regulating activity-dependent release of native brain-derived neurotrophic factor from hippocampal neurons. J Neurosci 22: 10399–10407. 12451139 10.1523/JNEUROSCI.22-23-10399.2002 PMC6758764

[19] Minichiello L (2009) TrkB signalling pathways in LTP and learning. Nat Rev Neurosci 10: 850–860. 19927149 10.1038/nrn2738

[20] Kandel ER (2004) The molecular biology of memory storage: a dialog between genes and synapses. Biosci Rep 24: 475–522. 16134023 10.1007/s10540-005-2742-7

[21] Malenka RC, Bear MF (2004) LTP and LTD: an embarrassment of riches. Neuron 44: 5–21. 15450156 10.1016/j.neuron.2004.09.012

[22] Huang EJ, Reichardt LF (2003) Trk receptors: roles in neuronal signal transduction. Annu Rev Biochem 72: 609–642. 12676795 10.1146/annurev.biochem.72.121801.161629

[23] Haapasalo A, Sipola I, Larsson K, Akerman KE, Stoilov P, et al (2002) Regulation of TRKB surface expression by brain-derived neurotrophic factor and truncated TRKB isoforms. J Biol Chem 277: 43160–43167. 12202482 10.1074/jbc.M205202200

[24] Yamada K, Nabeshima T (2003) Brain-derived neurotrophic factor/TrkB signaling in memory processes. J Pharmacol Sci 91: 267–270. 12719654 10.1254/jphs.91.267

[25] Perera FP, Tang D, Tu YH, Cruz LA, Borjas M, et al (2004) Biomarkers in maternal and newborn blood indicate heightened fetal susceptibility to procarcinogenic DNA damage. Environ Health Perspect 112: 1133–1136. 15238289 10.1289/ehp.6833 PMC1247389

[26] Hudon L, Moise KJ Jr, Hegemier SE, Hill RM, Moise AA, et al (1998) Long-term neurodevelopmental outcome after intrauterine transfusion for the treatment of fetal hemolytic disease. Am J Obstet Gynecol 179: 858–863. 9790359 10.1016/s0002-9378(98)70178-4

[27] Wormley DD, Chirwa S, Nayyar T, Wu J, Johnson S, et al (2004) Inhaled benzo(a)pyrene impairs long-term potentiation in the F1 generation rat dentate gyrus. Cell Mol Biol (Noisy-le-grand) 50: 715–721. 15641162

[28] Perera FP, Rauh V, Whyatt RM, Tsai WY, Tang D, et al (2006) Effect of prenatal exposure to airborne polycyclic aromatic hydrocarbons on neurodevelopment in the first 3 years of life among inner-city children. Environ Health Perspect 114: 1287–1292. 16882541 10.1289/ehp.9084 PMC1551985

[29] Sarsour K, Sheridan M, Jutte D, Nuru-Jeter A, Hinshaw S, et al (2011) Family socioeconomic status and child executive functions: the roles of language, home environment, and single parenthood. J Int Neuropsychol Soc 17: 120–132. 21073770 10.1017/S1355617710001335

[30] Wormley DD, Ramesh A, Hood DB (2004) Environmental contaminant-mixture effects on CNS development, plasticity, and behavior. Toxicol Appl Pharmacol 197: 49–65. 15126074 10.1016/j.taap.2004.01.016

[31] Sheng L, Ding X, Ferguson M, McCallister M, Rhoades R, et al (2010) Prenatal polycyclic aromatic hydrocarbon exposure leads to behavioral deficits and downregulation of receptor tyrosine kinase, MET. Toxicol Sci 118: 625–634. 20889680 10.1093/toxsci/kfq304 PMC2984527

[32] Li Z, Chadalapaka G, Ramesh A, Khoshbouei H, Maguire M, et al (2012) PAH particles perturb prenatal processes and phenotypes: protection from deficits in object discrimination afforded by dampening of brain oxidoreductase following in utero exposure to inhaled benzo(a)pyrene. Toxicol Sci 125: 233–247. 21987461 10.1093/toxsci/kfr261 PMC3243744

[33] Toledo-Rodriguez M, Lotfipour S, Leonard G, Perron M, Richer L, et al (2010) Maternal smoking during pregnancy is associated with epigenetic modifications of the brain-derived neurotrophic factor-6 exon in adolescent offspring. Am J Med Genet B Neuropsychiatr Genet 153B: 1350–1354. 20583129 10.1002/ajmg.b.31109

[34] Gomez-Pinilla F, Vaynman S (2005) A “deficient environment” in prenatal life may compromise systems important for cognitive function by affecting BDNF in the hippocampus. Exp Neurol 192: 235–243. 15755541 10.1016/j.expneurol.2004.12.001

[35] Su TC, Chan CC, Liau CS, Lin LY, Kao HL, et al (2006) Urban air pollution increases plasma fibrinogen and plasminogen activator inhibitor-1 levels in susceptible patients. Eur J Cardiovasc Prev Rehabil 13: 849–852. 17001229 10.1097/01.hjr.0000219116.25415.c4

[36] Greenberg ME, Xu B, Lu B, Hempstead BL (2009) New insights in the biology of BDNF synthesis and release: implications in CNS function. J Neurosci 29: 12764–12767. 19828787 10.1523/JNEUROSCI.3566-09.2009 PMC3091387

[37] Yeh CM, Huang CC, Hsu KS (2012) Prenatal stress alters hippocampal synaptic plasticity in young rat offspring through preventing the proteolytic conversion of pro-brain-derived neurotrophic factor (BDNF) to mature BDNF. J Physiol 590: 991–1010. 22155932 10.1113/jphysiol.2011.222042 PMC3381323

[38] Cunha C, Brambilla R, Thomas KL (2010) A simple role for BDNF in learning and memory? Frontiers in Molecular Neuroscience 3.. 10.3389/neuro.02.001.2010 PMC2821174 20162032

[39] Neeley EW, Berger R, Koenig JI, Leonard S (2011) Prenatal stress differentially alters brain-derived neurotrophic factor expression and signaling across rat strains. Neuroscience 187: 24–35. 21497180 10.1016/j.neuroscience.2011.03.065 PMC3114039

[40] Friedman WJ (2000) Neurotrophins induce death of hippocampal neurons via the p75 receptor. J Neurosci 20: 6340–6346. 10964939 10.1523/JNEUROSCI.20-17-06340.2000 PMC6772976

[41] Martinowich K, Hattori D, Wu H, Fouse S, He F, et al (2003) DNA methylation-related chromatin remodeling in activity-dependent BDNF gene regulation. Science 302: 890–893. 14593184 10.1126/science.1090842

[42] Li H, Zhong X, Chau KF, Williams EC, Chang Q (2011) Loss of activity-induced phosphorylation of MeCP2 enhances synaptogenesis, LTP and spatial memory. Nat Neurosci 14: 1001–1008. 21765426 10.1038/nn.2866 PMC3273496

[43] Chen WG, Chang Q, Lin Y, Meissner A, West AE, et al (2003) Derepression of BDNF transcription involves calcium-dependent phosphorylation of MeCP2. Science 302: 885–889. 14593183 10.1126/science.1086446

